# Probiotics as Adjuvants to Standard *Helicobacter pylori* Treatment: Evidence for the Use of Lacidofil^®^, an Established Blend of Thoroughly Characterized Strains

**DOI:** 10.3390/microorganisms13102223

**Published:** 2025-09-23

**Authors:** Noémie Auclair-Ouellet, Annie Tremblay, Ola Kassem, Sara E. Caballero-Calero, Stéphane Bronner, Sylvie Binda

**Affiliations:** Rosell Institute for Microbiome and Probiotics, 6100 Royalmount Avenue, Montreal, QC H4P 2R2, Canada; nauclairouellet@lallemand.com (N.A.-O.); atremblay@lallemand.com (A.T.); okassem@lallemand.com (O.K.); scaballero@lallemand.com (S.E.C.-C.); sbronner@lallemand.com (S.B.)

**Keywords:** *Helicobacter pylori* (*H. pylori*), treatment, probiotics, *Lacticaseibacillus rhamnosus* Rosell^®^-11, *Lactobacillus helveticus* Rosell^®^-52

## Abstract

*Helicobacter pylori* (*H. pylori*) is a bacterial pathogen that infects half of the world population. While standard treatment was initially effective, eradication rates have declined over the last 20 to 30 years, and the use of adjuvants, such as probiotic supplements, has been suggested to improve efficacy. This review presents evidence supporting the use of Lacidofil^®^, an established blend of two thoroughly characterized probiotic strains, as an adjuvant to standard therapy for *H. pylori* eradication. The microbiology and epidemiology of *H. pylori* infection as well as current approaches to diagnosis and treatment are summarized, and the roles of probiotics to support standard *H. pylori* treatment are outlined. Lacidofil^®^ and its component strains are described, and evidence from eight clinical trials supporting its efficacy is presented. *H. pylori* eradication rates were increased in participants receiving Lacidofil^®^ (90–100%) compared to controls (70–86.7%), and the incidence of side effects was decreased (e.g., antibiotic-associated diarrhea—Lacidofil^®^: 0–13.6%; controls: 20–40.9%). Published summaries, including systematic reviews with meta-analysis and an umbrella review, are discussed. To expand on the discussion of clinical studies, in vivo and in vitro studies are reviewed, including studies using state-of-the-art molecular methods. They characterize Lacidofil^®^’s mechanism of action and further support its efficacy as an adjuvant strategy for *H. pylori* eradication, side effect reduction, and return to gut microbiota homeostasis.

## 1. Introduction

*Helicobacter pylori* (*H. pylori*) is a bacterial pathogen that infects about half of the world population [1]. While most carriers remain asymptomatic, and therefore untreated, long-term *H. pylori* infection is associated with several conditions including chronic gastritis, ulcers, and cancer [1,2,3]. Therapies for *H. pylori* eradication were initially effective, but eradication rates have declined steadily over the last 20 to 30 years. Therefore, the use of adjuvants, such as probiotic supplements, has been suggested to improve efficacy [4,5].

Probiotics are defined as “live microorganisms that, when administered in adequate amounts, confer a health benefit on the host” [6]. For over 20 years, they have been used as an adjunctive solution to standard therapy for *H. pylori* eradication. Their efficacy for the improvement of eradication rates and reduction of side effects of standard therapy has been supported by dozens of clinical trials, whose results have been aggregated and summarized in systematic reviews and meta-analyses (see Yang et al. [7] for an umbrella review of systematic reviews with meta-analysis). Yet, uncertainty remains regarding probiotics’ efficacy, their role in supporting standard treatment, and the choice of the most effective probiotic solution for the specific purpose of *H. pylori* eradication [8,9,10,11,12]. This appears to be related to limitations in studies’ design and variability in probiotic strains or blends used in different studies, which makes the identification of clear conclusions and recommendations difficult [8,9,10,11,12]. In fact, there is growing consensus that the efficacy of probiotics needs to be evaluated on a strain-specific or blend-specific basis, considering the mounting evidence for the specific effects of different strains from the same genus or species on host health [13,14]. Moreover, the lack of mechanistic evidence for specific probiotic strains and blends represents an additional hurdle in the selection of the most appropriate probiotic solution [15,16]. However, some probiotics have been used for the purpose of supporting *H. pylori* standard treatment in several clinical studies and have been studied using diverse in vivo and in vitro methodologies that help uncover their mechanisms of action. A comprehensive synthesis of those studies can bring a meaningful contribution to the field of probiotic adjuvants in gastrointestinal therapy.

The aim of this narrative review is to present both clinical and mechanistic evidence in support of a probiotic-based adjuvant to *H. pylori* treatment. More specifically, the review presents evidence supporting the use of Lacidofil^®^, an established blend of two thoroughly characterized probiotic strains, as an adjuvant to increase *H. pylori* eradication rates and reduce side effects associated with standard therapy. In the first part, the microbiology and epidemiology of *H. pylori* infection as well as current approaches to diagnosis and treatment are summarized, and the roles of probiotics to support standard *H. pylori* treatment are presented. Then, Lacidofil^®^ and its component strains are described, and a summary of clinical evidence supporting its efficacy as an adjuvant to *H. pylori* standard treatment and preclinical studies demonstrating its mechanism of action is presented.

### Literature Search

Clinical studies included in this review have been selected based on their use of the Lacidofil^®^ probiotic blend as an adjuvant to *H. pylori* standard treatment, with no restriction with respect to the age of participants, the date of publication, the country where the study was conducted, or the standard *H. pylori* treatment used. Preclinical studies elucidating the probiotic supplement’s mechanisms of action have been selected based on their focus on the Lacidofil^®^ blend and/or its component strains, with no restriction with respect to the model or methodological approach used (e.g., in vitro and in vivo studies; murine or other rodent models), and the date of publication. Studies have been identified through keyword searches on scientific literature databases (PubMed, Google Scholar) and by screening reference lists from identified articles.

## 2. Microbiology and Epidemiology of *H. pylori* Infection

*H. pylori* is a Gram-negative, spiral-shaped and flagellated bacterium which is urease-, catalase-, and oxidase-positive [2,3]. *H. pylori* has evolved several mechanisms that enable it to survive and grow in the highly acidic gastric environment that is hostile to most bacteria. Its urease activity creates a buffer that allows it to survive high acidity in the gastric lumen for a short time, while its shape and flagella contribute to its motility and its ability to cross the mucus layer and enter the more hospitable environment of the gastric mucosa [2,3,17]. There, it adheres to the gastric epithelium and induces the expression of proinflammatory factors, which lead to chronic gastritis and dysregulation of host immune response, further contributing to its long-term survival [1,18]. Additionally, most *H. pylori* strains express virulence factors that enhance their ability to colonize the gastric epithelium and increase pathogenicity [1,3].

Spiral-shaped bacteria growing in the human stomach had been observed and reported by pathologists in the late 19th and early 20th centuries, but *H. pylori* was only definitively identified in the early 1980s [2,3]. Soon after, its association with inflammation and related health conditions and diseases became evident [2]. Chronic gastric inflammation induced by *H. pylori* can lead to chronic gastritis, peptic ulcers, gastric ulcers, gastric cancer, and mucosa-associated lymphoid tissue (MALT) lymphoma [1]. It was classified as a type I carcinogen by the International Agency for Research on Cancer in 1994 and is considered as the most common cause of infection-related cancers [3]. In addition to its association with gastric cancer and MALT lymphoma, there is growing evidence that *H. pylori* infection is associated with an increased risk of colorectal cancer [19].

## 3. Diagnosis and Treatment of *H. pylori* Infection

*H. pylori* infection is typically contracted during childhood, but most carriers are asymptomatic and remain untreated [1]. In children, conditions that motivate testing for *H. pylori* infection include peptic ulcers, chronic idiopathic thrombocytopenic purpura, and intractable iron deficiency [10]. In adults, testing for *H. pylori* infection is performed in individuals with signs or symptoms suggestive of the infection or who have a history of *H. pylori*-related diseases [20]. Expanding testing to first-degree relatives and members of the household of people who have confirmed *H. pylori* infection or related diseases is recommended in recent guidelines [20].

There are several methods available to diagnose *H. pylori* infection, which can be divided into non-invasive (serology, urea breath test, stool antigen test), and invasive methods (histopathology, rapid urease test, bacteriological culture), the latter requiring the collection of biopsy specimens through endoscopy [1,21]. Recently, molecular testing using polymerase chain reaction (PCR) performed on stool or biopsy samples has been added to the array of tools available for the detection of *H. pylori* infection [1,20,21,22]. PCR can accurately diagnose infection and provide information on strains’ antibiotic susceptibility and resistance, thus potentially informing the most appropriate choice of treatment [20,22].

According to the 2024 guidelines from the American College of Gastroenterology (ACG), 14-day bismuth quadruple therapy, which combines a bismuth salt (e.g., bismuth sub-citrate or subsalicylate), a proton pump inhibitor (PPI), and two antibiotics (tetracycline and metronidazole) is the preferred *H. pylori* eradication treatment for treatment-naïve patients who have no proven antibiotic susceptibility [8,23]. In many parts of the world, triple therapy consisting of PPI and two antibiotics (often clarithromycin and amoxicillin) remains the most common first line of treatment [5,11,23,24]. Triple therapy was initially effective, but eradication rates have consistently declined over the past 20 to 30 years [4,25]. Aside from their limited efficacy, these treatments are expensive, complex, and associated with side effects, which compounds risks of poor compliance, treatment failure, and emergence of antibiotic resistance [26,27,28]. In this context, many have highlighted the need to consider the use of adjunctive solutions, such as probiotic supplements, to improve *H. pylori* treatment outcomes [4,5].

## 4. The Role of Probiotics

Probiotics can play two distinct yet complementary roles to support standard treatment of *H. pylori* infection: inhibit *H. pylori* activity and reduce side effects of standard therapy. Evidence from preclinical and clinical studies suggests that probiotic strains, particularly from the *Lactobacillus* genus, can inhibit *H. pylori* through various mechanisms. These strains have good two-hour survival rates in the gastric environment, which enables them to exert several functions [29]. *H. pylori* infection induces profound changes in the composition and diversity of the gut microbiota, which is further altered by standard treatment with antibiotics and PPIs [30,31]. Moreover, homeostasis is not necessarily restored after *H. pylori* eradication [31]. This loss of homeostasis can be associated with an increased susceptibility to infection and vulnerability to diseases. Probiotic supplements are commonly described as an effective strategy to help maintain and restore microbiota balance. In the case of *H. pylori* eradication, they could provide overall support and help replenish commensal bacteria of the gut microbiota, including *Lactobacillus* species, which are essential to ensure resilience against further infections [26,31].

**Figure 1:**
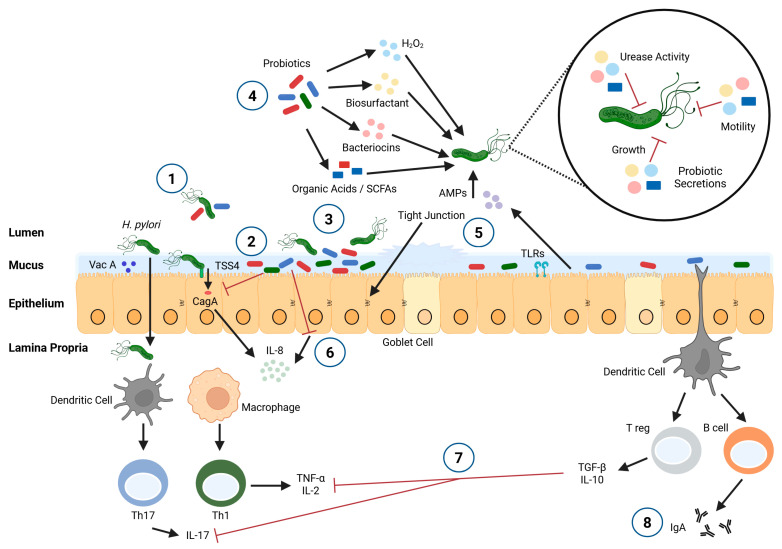
Proposed mechanisms of *H. pylori* inhibition by probiotics. (1) Prevention of *H. pylori* colonization by co-aggregation. (2) Adhesion to receptor sites on gastric epithelial cells (competitive exclusion). (3) Disruption/destruction of biofilm. (4) Production of organic acids that disrupt urease activity, as well as other antibacterial substances (hydrogen peroxide, bacteriocins, biosurfactants), and induction of antimicrobial peptide production by epithelial cells. (5) Stimulation of mucin synthesis, which contributes to the protection of tissues, and production of short-chain fatty acids (SCFAs), which strengthen tight junctions and support gastrointestinal barrier integrity. (6) Inhibition of IL-8 production, including through the alteration of CagA delivery to host cells by *H. pylori*. (7) Modulation of pro- (e.g., TNF-α) and anti-inflammatory (e.g., IL-10) cytokines, resulting in reduced inflammation. (8) increased production of immunoglobulin A (IgA). AMPs: antimicrobial peptides; CagA: cytotoxin-associated gene A; H_2_O_2_: hydrogen peroxide; IL: interleukin; SCFAs: short-chain fatty acids; TGF-β: transforming growth factor beta; Th: helper T cell; TLRs: toll like receptors; TNF-α: tumor necrosis factor alpha; Treg: regulatory T cell; TSS4: type IV secretion system; Vac A: vacuolating cytotoxin A. Created with BioRender.com.

Specific mechanisms by which probiotics inhibit *H. pylori* activity have not been fully elucidated to date, but they include (see Figure 1) (1) prevention of *H. pylori* colonization by co-aggregation, (2) adhesion to receptor sites on gastric epithelial cells (competitive exclusion), (3) disruption/destruction of biofilm, (4) production of organic acids that disrupt urease activity, as well as other antibacterial substances (hydrogen peroxide, bacteriocins, biosurfactants), and induction of antimicrobial peptide production by epithelial cells, (5) stimulation of mucin synthesis, which contributes to the protection of tissues, and production of short-chain fatty acids (SCFAs), which strengthen tight junctions and support gastrointestinal barrier integrity, (6) inhibition of IL-8 production, including through the alteration of CagA delivery to host cells by *H. pylori*, (7) modulation of pro- (e.g., TNF-α) and anti-inflammatory (e.g., IL-10) cytokines, resulting in reduced inflammation, and (8) increased production of immunoglobulin A (IgA) [14,16,29,31,32]. Evidence suggests that probiotics taken alone diminish pathogen load, while probiotics taken in combination with standard therapy improve eradication rates and alleviate side effects [31].

Probiotics’ ability to alleviate side effects of standard therapy (e.g., diarrhea, nausea/vomiting, gastric discomfort, bloating/gas, pain, constipation, taste disturbance) represents another significant contribution to the treatment of *H. pylori* infection. Patients who experience these symptoms may be tempted to skip standard therapy or stop it before full completion, which compromises treatment success [16,27,28]. In fact, lack of patient compliance has been identified as one of the main causes of *H. pylori* eradication failure [27,28]. Not only does poor compliance negatively affect the efficacy of treatment, but it may lead to recurrent infections, complications, and resistance to antibiotics [16,27,28,33]. Probiotics have been known to reduce side effects associated with standard therapy, including antibiotic-associated diarrhea (AAD), and they have been used as an adjuvant to standard treatment for various indications [34,35].

The evaluation of probiotics’ efficacy as adjuvants to standard *H. pylori* treatment is complicated by the diversity of strains and blends used in clinical trials, as well as differences in study designs, populations, standard treatment used (triple or quadruple therapy; specific medications used), and other factors [8,9,10,11]. While bacteria belonging to a specific genus or species share key characteristics and properties, there is growing consensus on the need to evaluate the efficacy of probiotics at the strain-specific level [13,14]. The following sections will review the evidence supporting the efficacy of an established probiotic blend, Lacidofil^®^.

## 5. Lacidofil^®^

Available since 1995, Lacidofil^®^ is a blend of two probiotic strains, *Lacticaseibacillus rhamnosus* Rosell^®^-11 and *Lactobacillus helveticus* Rosell^®^-52, at a ratio of 95:5. *L. rhamnosus* Rosell^®-^11 was isolated from a dairy starter culture in 1976 and deposited as I-1720 at the Institut Pasteur Collection Nationale de Cultures de Microorganismes (CNCM, Paris, France). Prior to the reclassification of the *Lactobacillus* genus in 2020 [36], this strain was identified as *Lactobacillus rhamnosus*. *L. helveticus* Rosell^®^-52 was isolated from a dairy starter culture in 1990 and deposited as I-1722 at the Institut Pasteur CNCM. Based on its biochemical and metabolic activity, it was initially identified as *Lactobacillus acidophilus* but was later reclassified as *L. helveticus* [37,38]. Both strains have been comprehensively characterized through genomic sequencing [39,40] and in vitro and in vivo studies. Their safety and that of their corresponding species is supported by their inclusion in lists and monographs on the safe use of probiotic substances [41,42,43]. Lacidofil^®^ has been approved as a medicinal product in some countries since 1995 and has been the object of a pharmacovigilance program since then. Owing to its safety and efficacy, it has received approval from Health Canada for the following health claims: restores and normalizes the intestinal flora; helps maintain a healthy microbiota in adults; helps maintain a healthy microflora in adults and pregnant women who are undergoing antibiotic therapy; helps reduce risks of AAD in children and adolescents aged 2 to 18; helps reduce the incidence of AAD in children and adolescents aged 6 to 16 who are undergoing antibiotic therapy for *H. pylori* infection; is an adjunct to physician-supervised therapy in patients with *H. pylori* infection; reduces the duration of acute infectious diarrhea in adolescents and adults aged 13 and older.

## 6. Clinical Studies

Eight studies published between 2002 and 2023 have assessed the efficacy of Lacidofil^®^ as an adjuvant to *H. pylori* therapy [44,45,46,47,48,49,50,51]. Study characteristics are presented in Table 1. 

### 6.1. Studies Conducted in Children

Three studies have investigated Lacidofil^®^ as an adjuvant solution for *H. pylori* eradication in children [44,45,46]. Plewinska et al. studied the effects of probiotics on *H. pylori* eradication, gastroduodenal inflammation, and incidence of side effects [44]. The study included 60 children who were diagnosed endoscopically with a rapid urease test confirmed through histological examination. Children were divided into two equal groups that received standard therapy with Lacidofil^®^ or with a placebo. They took standard therapy with the probiotic or placebo for 10 days, and the probiotic or placebo intervention alone for an additional 20 days. *H. pylori* eradication was confirmed by both negative urease test and negative histological examination six weeks after treatment completion. The eradication rate was significantly higher in the probiotic group (100%) than the placebo group (76.6%, *p* < 0.05). In addition, more children in the probiotic group were free of gastroduodenal lesions at the end of treatment compared to children in the placebo group (66.7% vs. 33.6%, *p* < 0.05). The prevalence of active chronic gastritis and duodenitis was significantly lower in children in the probiotic group compared to children in the placebo group (gastritis: 10% vs. 56.7%, *p* < 0.05; duodenitis: 0% vs. 33.3%, *p* < 0.05). Lastly, abdominal pain, taste disturbance, nausea/vomiting, and diarrhea were all significantly less prevalent (all *p* values < 0.05) in the probiotic group than the placebo group.

Gnaytenko et al. were interested in the effects of probiotics on the prevention of AAD in children receiving standard therapy for *H. pylori* eradication [45]. They recruited 25 children who received Lacidofil^®^ in addition to the standard treatment regimen, and 20 children who received standard therapy only and acted as controls. The infection was diagnosed with histological examination of gastroduodenal biopsy samples, and *H. pylori*-antigen test of feces. AAD was more frequent in children in the control group (7/20) than children in the probiotic group (2/25). Among children with AAD, more children in the control group (5/7) tested positive for *Clostridioides difficile* toxins A and B than children in the probiotic group (1/2). Eradication rates are not reported, but the authors mention that therapy had to be stopped in one child in the control group, while it was completed in all children in the probiotic group.

Abaturov and Gerasymenko studied the effects of Lacidofil^®^ on infection symptoms, side effects, and eradication rates [46]. In their study, 25 children received standard therapy for one week and probiotics for three weeks. They were compared to a group of 20 children who received standard therapy only for one week. All children underwent endoscopic examination and abdominal ultrasound. *H. pylori* infection was confirmed by rapid urease test, urease breath test, and serology. Authors observed that children who received the probiotic experienced more rapid relief of disease symptoms, such as abdominal pain and dyspeptic symptoms. Two children in the probiotic group experienced side effects compared to one third of children in the control group. At follow-up six weeks after treatment completion, *H. pylori* eradication was confirmed in 96% of children in the probiotic group compared to 70% of children in the control group (*p* < 0.05).

In summary, two studies report that Lacidofil^®^ increased eradication rates by approximately 25% compared to standard therapy alone. Positive results on the reduction of side effects were reported across studies, with two studies showing significantly lower prevalence in children taking Lacidofil^®^.

### 6.2. Studies Conducted in Adults

Five studies have investigated Lacidofil^®^ as an adjuvant solution for *H. pylori* eradication in adults [47,48,49,50,51]. Bielanski et al. evaluated the efficacy of standard triple therapy supplemented with probiotics [47]. They recruited 150 adults with *H. pylori* infection confirmed endoscopically through histology and rapid urease test, and with urease breath test. Fifty-one (51) participants received Lacidofil^®^ in addition to standard therapy. Probiotics were initiated with the standard therapy and taken together for 10 days, and then alone for an additional 10 days. Eradication rates confirmed through urease breath test four weeks after the end of treatment were significantly increased in the probiotic group (92%) compared to the control group (72%) (OR = 4.63; 95% CI: 1.42–16.73). In the probiotic group, 16% of participants reported slight taste disturbance, while 37% of control participants reported moderate taste disturbance and/or diarrhea, which caused one participant to leave the study before the end.

Ziemniak studied several questions related to the efficacy of standard therapy for *H. pylori* eradication [48]. The study included several arms comprising participants receiving different treatment regimens, including 53 participants who received standard treatment (PPI and antibiotics) supplemented with Lacidofil^®^. These participants had gastric or duodenal ulcers identified through endoscopy, chronic gastritis, and *H. pylori* infection confirmed with urease breath test. At the end of treatment, the eradication rate (confirmed by urease breath test) was higher in the probiotic group (94.3%) compared to the standard treatment group (85.9%, *p* < 0.05).

Babak assessed the efficacy and safety of standard therapy supplemented with Lacidofil^®^ in individuals with duodenal ulcers due to *H. pylori* infection [49]. All participants completed clinical examination, serology, and endoscopic examination with collection of biopsy samples that were analyzed histologically. *H. pylori* eradication was confirmed four weeks after the end of treatment through endoscopic examination and histological analysis, rapid urease test, and normalization of the gastrointestinal microbiota. All participants received standard therapy for seven days. Participants in the probiotic group also took Lacidofil^®^ for the full course of standard therapy, and 13 more days (20 days in total). Abdominal pain and dyspeptic symptoms were resolved faster in the probiotic group than the control group. The eradication rate was slightly higher in the probiotic group (90%) than the control group (86.7%). The abundance of *Bifidobacteria* and *Lactobacilli* increased, and the abundance of opportunistic pathogens decreased in the probiotic group.

Vdovychenko et al. compared the efficacy of standard therapy to the efficacy of therapy supplemented with probiotics in participants with duodenal ulcers associated with *H. pylori* infection [50]. Duodenal ulcers were assessed through endoscopy. This examination was repeated four weeks after the end of treatment, along with confirmation of *H. pylori* eradication through rapid urease test, urease breath test, cell culture, and antigen test from feces samples. One participant (4%) in the probiotic group experienced side effects compared to six participants (25%) in the control group. At follow-up, healing of ulcerative lesions was observed in 88% of participants in the probiotic group compared to 70.8% of participants in the control group. *H. pylori* eradication was achieved in 96% of participants in the probiotic group compared to 75% of participants in the control group.

Lastly, Kiattiweerasak et al. tested the efficacy of a 14-day standard triple therapy supplemented with Lacidofil^®^ to the same therapy supplemented with a placebo in participants who had not completed previous *H. pylori* treatment [51]. This recent study was conducted in accordance with the International Council for Harmonisation of Technical Requirements for Pharmaceuticals for Human Use guideline for Good Clinical Practice (ICH-GCP). *H. pylori* infection was confirmed with biopsies from upper gastrointestinal endoscopy that were analyzed with rapid urease test, *H. pylori* culture, and histology. Participants took the probiotic or placebo for the full course of standard therapy (14 days) and an additional 28 days (42 days in total). According to the per protocol analysis, probiotic supplementation provided a significantly higher eradication rate than the placebo (90.9% vs. 75.0%, *p* < 0.05). Gastrointestinal symptoms assessed with the Gastrointestinal Symptom Rating Scale (GSRS) were significantly improved in the probiotic group compared with those of the placebo group after the course of standard therapy (14 days) (2.31 ± 0.45 vs. 2.91 ± 0.70, *p* < 0.001), and after full completion of the probiotic or placebo course (42 days) (1.46 ± 0.36 vs. 2.65 ± 0.66, *p* < 0.001). Health-related quality of life was also significantly improved in the probiotic group compared to the placebo group after 42 days (63.3 ± 10.2 vs. 57.3 ± 13.4, *p* = 0.020). Additionally, participants in the probiotic group had a significantly lower incidence of side effects compared to participants in the placebo group, including bloating (15.9% vs. 40.9%; OR 0.27 [95% CI 0.10 to 0.75], *p* = 0.012), diarrhea (13.6% vs. 40.9%; OR 0.23 [95% CI 0.28 to 0.65], *p* = 0.006), taste disturbance (4.5% vs. 25.0%; OR 0.14 [95% CI 0.03 to 0.69], *p* = 0.015), and nausea (2.3% vs. 34.1%; OR 0.05 [95% CI 0.01 to 0.36], *p* = 0.003). None of the participants experienced serious adverse events.

In summary, probiotic supplementation was associated with increased eradication rates compared to standard treatment alone. In studies reviewed, eradication rates in adult participants taking standard treatment supplemented with Lacidofil^®^ ranged from 90% [49] to 96% [50]. In comparison, eradication rates in participants receiving the standard treatment alone or with a placebo ranged from 72% [47] to 86.7% [49]. In other words, eradication rates in participants receiving standard therapy supplemented with Lacidofil^®^ reached the 90% eradication threshold in all five studies, while none of the standard treatment alone or with a placebo did. In addition, more rapid resolution of symptoms and increased improvement of symptoms compared to the placebo were reported, along with reduced incidence of side effects.

### 6.3. Summary of Clinical Studies’ Characteristics

Many studies across age groups had a small sample. However, they reported complete results or clearly provided the number and reasons for participant dropouts. Three studies reported implementing methods to randomly assign participants to interventions [46,49,51], and two used a placebo [44,51]. Except for the recent study by Kiattiweerasak et al. [51], descriptions of the experimental design lacked details. It is unlikely that studies that did not use a randomized controlled design implemented measures to blind participants and study personnel to intervention allocation. Nevertheless, they reported results for prespecified outcomes, and exploratory analyses were clearly presented as such.

The age range was large in some studies, which could imply interindividual differences in infection duration and stage. Using a narrower age range or recruiting larger groups of participants to perform subgroup analyses could have helped address this limitation. Related to the question of age range, probiotic dosage was lower in studies conducted in children but could have been adjusted for adolescents to make it more similar to the dosage used in adults. Most studies across age groups provided Lacidofil^®^ supplements for periods ranging from 20 to 30 days. However, Vdovychenko et al. [50] obtained similar results both in terms of enhancement of eradication rates and reduction of side effects using 10-day supplementation. The duration of standard therapy and medications used were similar across studies, but some differences are to be expected considering changes in practices and guidelines that occurred over the time they were conducted. Overall, while these studies present some limitations and no two studies used identical designs, consistency in results supports the efficacy of Lacidofil^®^ as an adjuvant to standard treatment for *H. pylori* eradication.

### 6.4. Inclusion of Studies in Systematic Reviews and Meta-Analyses

Several of the studies reviewed above have been included in systematic reviews and meta-analyses focused on the evaluation of probiotic supplementation efficacy to support standard treatment for *H. pylori* eradication and to alleviate common side effects [52,53,54,55,56,57,58,59,60,61,62,63,64]. Those reviews provide a summary of evidence for various probiotic strains and blends, including different commercially available formulations. Among those reviews and meta-analyses, some stand out for their approach of grouping studies that use the same strain or the same blend of strains [58,59,60,62], which allows for a direct comparison of different probiotic formulations and avoids biases and limitations of meta-analyses that aggregate results at the species or genus level [60,65].

Feng et al. [59] conducted a network meta-analysis of studies using probiotic supplementation as an adjuvant to standard triple therapy for *H. pylori* eradication in children. They identified 29 trials using 17 different probiotic solutions, including the study of Plewinska et al. [44]. Among all solutions compared, Lacidofil^®^ was the most effective at reducing overall side effects. McFarland et al. [58] conducted a meta-analysis of trials using multi-strain probiotic blends as a support to standard *H. pylori* treatment and identified 19 trials using 6 probiotic solutions. When considering pooled eradication rates, authors concluded that only Lacidofil^®^ and a blend of *L. acidophilus* and *B. animalis* contributed to high eradication rates when taken in combination with triple therapy. They also concluded that Lacidofil^®^ was effective at reducing overall side effects. In a 2018 meta-analysis, McFarland et al. assessed the strain-specific and disease-specific effects of different probiotic solutions [60]. They found that there was “strong evidence” in favor of Lacidofil^®^ as an effective adjuvant to *H. pylori* eradication. Lastly, in a 2021 meta-analysis comparing single strains to blends containing the same strains, McFarland et al. found that both *Lactobacillus helveticus* Rosell^®^-52 alone and combined with *Lacticaseibacillus rhamnosus* Rosell^®^-11 in the Lacidofil^®^ blend were protective against AAD in treatments for *H. pylori* eradication and other indications [62].

The abundance of systematic reviews and meta-analyses has generated a need for higher-level summaries that help describe the quality of evidence in each review, compare conclusions made across reviews, and evaluate the strength of these conclusions [66]. These summaries, called “reviews of reviews” or “umbrella reviews”, are aimed at supporting decision-making by summarizing and critically appraising large bodies of evidence. Yang et al. [7] recently published an umbrella review of systematic reviews with meta-analysis focused on the effects of probiotic supplementation on *H. pylori* standard treatment. They identified the meta-analysis of McFarland et al. [58] as the most comprehensive for the evaluation of the effects of multi-strain solutions. The evaluation of evidence provided by this umbrella review reinforces the conclusions of McFarland et al. [58] that Lacidofil^®^ increases eradication rates when combined with standard therapy, and that it reduces overall side effects.

## 7. Mechanisms of Action

While systematic reviews and meta-analyses are useful to compile evidence of probiotics’ efficacy, they rarely discuss their mechanisms of action. In vitro and in vivo studies of Lacidofil^®^ and its constituent strains shed light on the mechanisms by which they exert positive effects on people taking probiotic supplements. Their findings are coherent with the mechanisms underlying probiotics’ antagonizing effects on *H. pylori* and their protective effects against AAD. Mechanistic evidence for the Lacidofil^®^ probiotic blend and its component strains is summarized in Table 2.

### 7.1. In Vivo Studies

The study of Johnson-Henry et al. [67] was the first to investigate the effects of Lacidofil^®^ on *H. pylori* in vitro and in vivo. In this study, co-incubation of Lacidofil^®^ with *H. pylori* at a 1:1 ratio reduced colonization, while co-incubation at ratios of 1:10 and 1:100 inhibited colony formation. In mice undergoing *H. pylori* challenge, pretreatment and ongoing supplementation with Lacidofil^®^ reduced the rate of infection by half compared to the regular diet (*p* < 0.05). Furthermore, Lacidofil^®^ reduced the proportion of mice with moderate–severe gastric inflammation compared to the regular diet (29% vs. 71%). Brzozowski et al. [68] investigated several parameters of *H. pylori* infection in Mongolian gerbils treated with a vehicle, standard triple therapy, or Lacidofil^®^. Following infection, gastric acid, plasma gastrin, and luminal somatostatin levels were maintained by both standard therapy and Lacidofil^®^. Mucosal inflammation, gastric lesions, hyperplasia and apoptosis of gastric epithelial cells were eliminated by triple therapy and significantly reduced by Lacidofil^®^. Lastly, expression of proteins associated with inflammation (cyclooxygenase-2, COX-2) and apoptosis (BAX) was upregulated following infection, while expression of proteins associated with anti-apoptotic response (Bcl-2) was suppressed. Treatment with standard triple therapy eliminated these responses while supplementation with Lacidofil^®^ significantly attenuated them. Verdu et al. [70] were interested in the recovery of gastrointestinal function and feeding behavior in mice that had recently completed standard therapy for *H. pylori* eradication. They found that mice presented physiological changes, such as delayed gastric emptying and increased gastric immune cell (CD3+) counts, that persisted despite pathogen eradication. Those changes were associated with altered feeding behavior, whereby mice ate smaller amounts of food more frequently. Mice in which *H. pylori* had been eradicated were given either Lacidofil^®^, a placebo, or *H. pylori* antigen. Lacidofil^®^ accelerated the recovery of gastric emptying, improved CD3+ cell counts, and normalized feeding behavior. In contrast, antigen administration resulted in increased anti-*H. pylori* antibodies, increased CD3+ cell counts, and delayed normalization of gastric function.

### 7.2. In Vitro Studies on Lacidofil^®^ Strains

Recent studies on the strains included in Lacidofil^®^, *Lactobacillus helveticus* Rosell^®^-52 and *Lacticaseibacillus rhamnosus* Rosell^®^-11, provide important insights into their anti-inflammatory and antibacterial properties. In a study evaluating the properties of several lactic acid bacteria [16], *L. rhamnosus* Rosell^®^-11 reduced *H. pylori* urease activity (which is essential for *H. pylori* growth) and abrogated *H. pylori* motility even in the presence of urea. *L. helveticus* Rosell^®^-52 was efficient at curtailing *H. pylori* growth and showed significant urea resistance. It inhibited *H. pylori* motility and reduced *H. pylori*-induced elicitation of pro-inflammatory interleukin-8 (IL-8) secretion in the absence and presence of urea. Inflammation, and more specifically expression of IL-8, is considered a hallmark of *H. pylori* infection [16]. *H. pylori* is thought to fine-tune anti-inflammatory and pro-inflammatory responses to generate a state of chronic low-grade inflammation that is sufficient to cause tissue damage, enabling it to obtain nutrients, without triggering a level of inflammation that could compromise its own viability. This study shows that the strains that compose Lacidofil^®^ may inhibit *H. pylori* by reducing its infectivity (growth and motility) and disrupting *H. pylori*-induced inflammatory response in epithelial cells.

A series of studies [69,71,72,73,74] has investigated the immunomodulatory properties of *L. rhamnosus* Rosell^®^-11. *L. rhamnosus* Rosell^®^-11 was able to downregulate the lipopolysaccharide (LPS)-induced production of IL-8 by intestinal epithelial cells (cell line HT-29) and gastric epithelial cells (cell line KATO III) [71]. A subsequent study expanded on those results and showed that *L. rhamnosus* Rosell^®^-11 cell-free supernatant downregulated IL-8 production from HT-29 cells challenged with various Pattern Recognition Receptor (PRR) ligands and pro-inflammatory cytokines, and downregulated the production of CINC-1 induced by flagellin and LPS in a rat intestinal cell line (IEC-6) [72]. In more recent studies, the secretome of *L. rhamnosus* Rosell^®^-11 was shown to decrease the inflammatory response of human intestinal epithelial cells exposed to *Salmonella enterica* serovar Typhimurium secretome (STS) and TNF-α by increasing the transcription of negative regulators of innate immune activity and inducing changes in global H3 and H4 histone acetylation [73]. It induced secretion of macrophage inhibitory factor (MIF) and down-regulated pro-inflammatory mediator secretion resulting in attenuated CXCL8/IL-8 and NFκB1 expression. It also reversed damage to monolayer transepithelial resistance and permeability, a result that was confirmed through genetic analysis and was underpinned by MIF secretion [69]. Lastly, *L. rhamnosus* Rosell^®^-11 secretome induced a unique transcriptional profile in THP-1 monocytes, promoting macrophage differentiation and polarization and priming subsequent immune response by “training” the microbiota to shape immune response to pro-inflammatory challenges and preparing the host to respond to subsequent inflammatory signals [74].

In summary, those studies support the contribution of Lacidofil^®^ to the modulation of inflammatory and host immune response to *H. pylori* infection. They also support its effects on urease activity, which decreases *H. pylori* infectivity. Results from preclinical studies are in line with the findings of clinical studies, including the reduction of side effects and more rapid resolution of gastro-intestinal symptoms in participants taking probiotics, the positive effects of probiotics on gastric inflammation, lesions, and ulcers, and the increase in eradication rates, which could be mediated by probiotics’ effects on *H. pylori* viability and infectivity and modulation of inflammatory and immune responses. While these links between clinical and preclinical studies are coherent based on the evidence summarized in the current review, more studies, and especially studies using modern molecular methods, are needed to investigate the association between observed clinical benefits and their underlying mechanisms of action.

## 8. Limitations and Perspectives for Future Research

This review has some limitations. Clinical studies vary in their design and methodology, which is in part due to the standard *H. pylori* treatment that was used at the time and place where these studies were conducted. Moreover, only one of these studies was published in the last five years. Changes in recommendations and variability in specific treatment parameters (e.g., specific antibiotics used) represent a hurdle in the evaluation of efficacy, but they should not prevent from recognizing Lacidofil^®^’s positive effects on the enhancement of *H. pylori* eradication rates and reduction of side effects, which are reported in several studies and supported by systematic reviews with meta-analysis, and an umbrella review.

While the review summarizes the findings of recent in vitro studies, in vivo studies are more dated. These studies were included to provide a comprehensive review of evidence, but do not reflect state-of-the-art methodology. However, they used Mongolian gerbils and mice, which are still used as animal models of *H. pylori* infection despite their limitations (e.g., difficulties inducing chronic colonization, replicating disease stages, inducing the development of cancerous lesions, etc.) [75,76]. For instance, wild-type mice typically develop relatively mild gastritis and show a slow evolution of the disease, making them a less useful model [75]. The relevance of murine models can be improved by using genetically modified animals or animals that have been “humanized” with gut microbiota transplants [76]. On the other hand, Mongolian gerbils recapitulate many features of human disease (inflammation, ulceration, and carcinogenesis), and present similar symptoms (lack of appetite, weight loss), making them an appropriate and cost-effective model [75]. In general, the *H. pylori* strain and specific disease mechanisms under study should be carefully matched with a model that is suitable to answer specific research questions [75,76]. As in any field of investigation, in vitro and animal models represent approximations and simplifications of human physiology, and important differences should be acknowledged and considered when interpreting results [77,78].

The translation of findings from in vitro and in vivo studies to their application in clinical trials always represents a challenge, but the adoption of new technologies in the diagnosis and evaluation of treatment efficacy in the clinical management of *H. pylori* infection will likely contribute to bridge that gap [20,21,22]. For instance, PCR tests provide invaluable information on *H. pylori* strains and their properties (virulence, antibiotic susceptibility), which can be used to guide the most appropriate choice of treatment based on evidence coming from both clinical and fundamental research [20,21,22]. Preclinical studies investigating the effects of Lacidofil^®^ on various *H. pylori* strains could also contribute to elucidating its effects on specific virulence factors and may help further optimize interventions [1,3].

The adoption of new technologies and new approaches to the treatment of *H. pylori* infection (e.g., antibiotic stewardship) is expected to lead to more thorough monitoring of treatment efficacy [20] and may attract more attention to issues related to recovery from infection and return to gastrointestinal homeostasis, as well as prevention of reinfection. This is in line with recent recommendations to test immediate relatives and members of the household of people infected with *H. pylori*. Offering therapy to these individuals decreases their risks of developing complications and prevents reinfection [20]. With their ability to restore the balance of the gut microbiota, probiotics could play an important yet underexplored role in these aspects of *H. pylori* infection management [31]. Interestingly, beyond the increase of *H. pylori* eradication rates, Lacidofil^®^ has also shown positive effects on the healing of gastric inflammation, lesions, and ulcers both in vivo [67,68] and in clinical studies [44,50]. Mechanistic knowledge gained from studies focusing on *H. pylori* eradication can bring additional support for the use of Lacidofil^®^ to manage AAD associated with the treatment of other conditions, as well as for the maintenance and restoration of gut microbiota balance. Future clinical studies should use methods and approaches that help make clear connections between observed clinical benefits and probiotics’ mechanisms of action (e.g., multiomics approaches). Large multicenter trials with independent validation of data and use of contemporary quadruple therapy protocols would constitute a major contribution to knowledge. More specifically, using a multi-arm design with longer-term follow-up and focusing on the optimization of key parameters of probiotics regimens, such as optimal dosage, overall duration and timing with respect to standard therapy, could help further characterize Lacidofil^®^’s role and efficacy to support the treatment of *H. pylori* infection, and the prevention of other infections or reinfections. Considering geographical variations in the prevalence of *H. pylori* infection, local strains of *H. pylori* and associated virulence factors, and levels of resistance to different antibiotics, conducting studies in different parts of the world remains a priority [1,76,79,80].

## 9. Conclusions

Probiotics represent a relevant solution to support standard therapy for *H. pylori* eradication and reduce side effects of treatment, but not all strains and blends have the same mechanisms of action and present the same efficacy. This review advances the field of probiotic adjuvants in gastrointestinal therapy by summarizing evidence for the efficacy of Lacidofil^®^, an established probiotic solution composed of two well-characterized strains whose anti-inflammatory and immunomodulatory effects have been demonstrated in vitro and in vivo. Clinical studies have shown that Lacidofil^®^ increases *H. pylori* eradication rates and reduces side effects of standard therapy. Those conclusions have been supported by systematic reviews and meta-analyses, including one that was recently identified as the most comprehensive summary of evidence available to date for the use of probiotic blends to support standard *H. pylori* therapy. Lacidofil^®^ has also received approval from Health Canada for health claims related to *H. pylori* eradication treatment as well as maintenance and restoration of gut microbiota balance and gut health. As such, its application is not limited to a single condition and extends to other gastrointestinal indications. Future studies focusing on *H. pylori* eradication will further elucidate the contribution of Lacidofil^®^ as an adjuvant to standard therapy and will help refine treatment parameters to optimize efficacy.

## Figures and Tables

**Table 1:** Clinical studies investigating the effects of Lacidofil^®^ on *H. pylori* eradication.

First Author and Reference	Year	Country	Age (Years)	*n* Lacidofil^®^ Arm	*n* Control Arm	Lacidofil^®^ Regimen	Lacidofil^®^ Duration	Medications Used in Standard Therapy	Standard Therapy Duration	*H. pylori* Eradication ^a^	Frequency of Side Effects ^a^
Studies conducted in children											
Plewinska [44]	2006	Poland	8.8 to 18.3	30	30	2B 3x/day	30 days	Amoxicillin, clarithromycin, omeprazole	10 days	100 vs. 76.6 *	AAD: 0 vs. 20 * Abdominal pain: 3.3 vs. 23.3 * Nausea/vomiting: 0 vs. 20 * Taste disturbance: 3.3 vs. 23.3 *
Gnaytenko [45]	2009	Ukraine	6 to 16	25	20	2B 2x/day	20 days	Amoxicillin, clarithromycin	7 days	n.r.	AAD: 8 vs. 35 *
Abaturov [46]	2014	Ukraine	10 to 16	25	20	2B 3x/day	21 days	Amoxicillin, bismuth sub-citrate, clarithromycin	7 days	96 vs. 70 *	All SEs: 8 vs. 33.3 n.t.
Studies conducted in adults											
Bielanski [47]	2002	Poland	Median: 43	51	99	4B 3x/day	20 days	Amoxicillin, clarithromycin, pantoprazole	10 days	92 vs. 72 *	Taste disturbance and/or diarrhea: 16 (slight) vs. 37 (moderate/severe) n.t.
Ziemniak [48]	2006	Poland	18 to 81	53	192	4B 2x/day	20 days	Amoxicillin, clarithromycin, pantoprazole	10 days	94.3 vs. 85.9 *	n.r.
Babak [49]	2007	Ukraine	18 to 70	20	15	4B 3x/day	20 days	Amoxicillin, clarithromycin, rabeprazole	7 days	90 vs. 86.7 n.t.	n.r.
Vdovychenko [50]	2008	Ukraine	Mean: 43.9	25	24	4B 2x/day	10 days	Amoxicillin, clarithromycin, omeprazole	7 days	96 vs. 75 n.t.	All SEs: 4 vs. 25 n.t.
Kiattiweerasak [51]	2023	Thailand	18 to 65	45	45	4B 2x/day	42 days	Amoxicillin, clarithromycin, lansoprazole	14 days	90.9 vs. 75 *	AAD: 13.6 vs. 40.9 * Bloating: 15.9 vs. 40.9 * Nausea: 2.3 vs. 34.1 * Taste disturbance: 4.5 vs. 25 *

Legend: B: billion; n.r.: not reported; n.t.: not tested with inferential statistics; SE: side effect; AAD: antibiotic-associated diarrhea. ^a^: Percentage (%), Lacidofil^®^ vs. control. * Denotes a statistically significant difference.

**Table 2:** Mechanisms of action and related observations for the Lacidofil^®^ probiotic blend and its component strains.

Mechanism of Action	Probiotic	Observation	First Author and Reference
Pathogen inhibition	Lacidofil^®^	Co-incubation with *H. pylori* at a 1:1 ratio reduced colonization; co-incubation at ratios of 1:10 and 1:100 inhibited colony formation.	Johnson-Henry [67]
		In mice undergoing *H. pylori* challenge, reduced the rate of infection by half compared to the regular diet.	
	*L. rhamnosus* Rosell^®^-11	Reduced *H. pylori* urease activity, which negatively impacts *H. pylori* growth.	Whiteside [16]
		Abrogated *H. pylori* motility in the presence and absence of urea.	
	*L. helveticus* Rosell^®^-52	Curtailed *H. pylori* growth and showed significant urea resistance.	Whiteside [16]
		Inhibited *H. pylori* motility.	
Adhesion and gastrointestinal barrier integrity	Lacidofil^®^	In Mongolian gerbils infected with *H. pylori*, significantly reduced gastric lesions, as well as hyperplasia and apoptosis of gastric epithelial cells.	Brzozowski [68]
	*L. rhamnosus* Rosell^®^-11	Reversed damage to monolayer transepithelial resistance and permeability.	Jeffrey [69]
Inflammation and immune modulation	Lacidofil^®^	Reduced the proportion of mice with moderate–severe gastric inflammation compared to the regular diet.	Johnson-Henry [67]
		In mice that had completed *H. pylori* eradication treatment, improved gastric immune cell (CD3+) counts.	Verdu [70]
		In Mongolian gerbils infected with *H. pylori*, significantly reduced mucosal inflammation and gastric lesions.	Brzozowski [68]
		In Mongolian gerbils infected with *H. pylori*, attenuated the expression of proteins associated with inflammation (COX-2) and apoptosis (BAX).	
	*L. rhamnosus* Rosell^®^-11	Downregulated the LPS-induced production of IL-8 by intestinal epithelial cells (HT-29) and gastric epithelial cells (KATO III).	Wood [71]
		In pro-inflammatory challenges, downregulated IL-8 production by intestinal epithelial cells (HT-29) and CINC-1 production from a rat intestinal cell line (IEC-6).	Jeffrey [72]
		Decreased the inflammatory response of human intestinal epithelial cells exposed to *Salmonella enterica* serovar Typhimurium secretome (STS) and TNF-α.	Jeffrey [73]
		Induced secretion of macrophage inhibitory factor (MIF) and down-regulated pro-inflammatory mediator secretion resulting in attenuated CXCL8/IL-8 and NFκB1 expression.	Jeffrey [69]
		Induced a unique transcriptional profile in THP-1 monocytes, promoting macrophage differentiation and polarization and priming subsequent immune response.	Jeffrey [74]
	*L. helveticus* Rosell^®^-52	Reduced *H. pylori*-induced elicitation of pro-inflammatory IL-8 secretion in the absence and presence of urea.	Whiteside [16]

## Data Availability

No new data were created or analyzed in this study. Data sharing is not applicable to this article.
